# Incidence of PTSD and generalized anxiety symptoms during the first wave of COVID-19 outbreak: an exploratory study of a large sample of the Italian population

**DOI:** 10.1186/s12889-021-11168-y

**Published:** 2021-06-16

**Authors:** Eleonora Brivio, Serena Oliveri, Paolo Guiddi, Gabriella Pravettoni

**Affiliations:** 1 Applied Research Division for Cognitive and Pychological Science, Istituto Europeo d’Oncologia IRCCS, Viale Ripamonti 435, 20141 Milan, Italy; 2 Department of Oncology and Hemato-Oncology, University of Milan, Milan, Italy

**Keywords:** Post-traumatic stress disorder, Generalized anxiety, COVID-19

## Abstract

**Background:**

During the COVID-19 pandemic, between March and May 2020, in Italy, people were asked to shelter in place and most had to put their life on hold, while news of the spread of the virus constantly were broadcasted. The change in habits and the potential exposure to a dangerous virus can be categorized as a catastrophic event, which are usually traumatic and therefore have psychological consequences for the people involved.

**Objective:**

Assuming the COVID-19 pandemic as a possible traumatic event, this study aims to explore the contingent behavioural and psychological impact of COVID-19 spread and related lockdown on the Italian general population, through measuring anxiety and post-traumatic stress symptoms .

**Methods:**

An ad hoc-survey was set up for the this exploratory research, including the standardized Italian versions of the Impact of Event Scale Revised (IES-R) and the Generalized Anxiety Scale (GAD-7), and ad hoc items concerning behavioural reactions due to the pandemic spread and related mass quarantine. Participants were recruited across convenient web-based and mobile app channels using a snowball sampling technique. Data was collected from March 25th to May 1st, 2020.

**Participants:**

One-thousand one hundred and ninety-five individuals (851 women and 342 men) completed 60% or more of the survey and were considered for analyses. Mean age was 40 years (s.d. = 14.948). Participants resided in 78 Italian provinces (out of 107 territorial divisions), with 25.7% residing in the Milan province and 9.2% from the Monza and Brianza provinces, closest to the epicentre of the Italian outbreak.

**Results:**

During the worst months of the first wave of the Pandemic, the Italian population suffered high level of distress (GAD-7 m = 6.89, s.d. = 5.08; IER-R mean score = 27.86, s.d. 17.46), respectively indicating mild presence of anxiety symptoms, and high levels of PTSD symptoms. Gender seems to be a discriminating variable with women scoring significantly higher than man, both for anxiety symptoms (H (1) = 82.91, *p* < .001) and all dimensions of PTSD symptoms (intrusion H (1) = 71.23, *p* < .001, avoidance H (1) = 61.28, p < .001), and hyperarousal (H (1) = 67.348, p < .001). People from Generations Y and Z show to be at higher risk of developing PTSD (V = .746, F (3,1041) =1017.19, *p* = .001) and GAD symptoms (F (3,1041) = 5.113, *p* = .002) than older generations.

**Conclusions:**

Gender and generation appeared to be the most consistent risk factor for higher levels of generalized anxiety and PTSD symptoms in the current pandemic. Other variables – such as information seeking behaviours, parental and marriage status, chronic conditions – yielded less consistent evidence. Results indicate the need of including psychological interventions as a standard tool during the emergency management of a catastrophic events such as a pandemic.

## Background

The COVID-19 pandemic originated in Wuhan, China and spread to Europe in January 2020. Among these EU countries, Italy was one of the countries most severely struck by the pandemic. After two cases in Rome as of January 31st and another in Codogno on February 6th, by February 21st, the number of cases were rapidly increasing in Lombardy and Veneto regions [1]. Despite feelings of discouragement, fear, restriction, and generalized impotence resulting from the media’s constant reporting of increasing numbers of infected individuals and associated deaths, Italy rose to the occasion and quickly became a key leader in the fight against the virus [2, 3].

Between March April 2020, the mortality rate in Italy increased from four to 8 % [4]. Newspapers, social media, and television began circulating images of the imminent catastrophe, with military wagons carrying the coffins of Covid-19 victims in Bergamo, Lombardy, and for individuals within the population who had been quarantined, such images triggered further panic and perceptions of having no escape [5, 6]. The spread of an infectious disease like the Covid-19 can be considered a type of catastrophic event, such as hurricanes, floods, and earthquakes, and similar psychological consequences can result [7, 8]. A meta-analysis of literature examined the correlation between mental health and catastrophic events [9], finding that about one-third of these studies reported an important effect on the state of the psychological well-being of the populations. The most common diagnoses were, in order of incidence, Post-Traumatic Stress Disorder (PTSD), Major Depression, and Generalized Anxiety Disorder (GAD). Severe Acute Respiratory Syndrome (SARS), was the first new infectious disease of the twenty-first century and with its spread, stress, stigma, and anxiety became common manifestations among infected individuals [10, 11]. In 2006, results from research conducted on 129 people quarantined in Toronto during the spread of SARS showed an incidence of 31.2% for Depression and 28.9% for PTSD [12].

COVID-19 pandemic aggressiveness is burdening more than the previous SARS endemic [13–15]. The study conducted by Wang et al. [16] explored the impact of the COVID-19 pandemic on both the physical and the mental health of Chinese citizens through an online questionnaire. Results showed that 53.8% of respondents reported a moderate to severe impact from the pandemic on their psychological well-being. More specifically, 16.5% reported moderate to severe depressive symptoms, 28.8% reported moderate to severe anxious symptoms, and 8.1% reported moderate to severe stress levels. Females, students, and those with physical symptoms like muscle pain, coldness, and dizziness were the most at risk of psychological vulnerability. In contrast, predictors of greater psychological well-being included frequently and carefully following the latest updates regarding the pandemic and adopting precautionary measures like hand hygiene and use of masks [17, 18].

Current literature suggests that the COVID-19 pandemic can create a consequent anxiety pandemic [13, 19] and many studies provide evidence of PTSD symptoms in different populations. Fekih-Romdhane et al. (2020) investigated incidence of PTSD symptoms in a sample of Tunisian citizens, and found that 33.0% of participants scored higher than 33 (threshold cut-off) on IES-R scale, with “avoidance” as the most frequent symptom, and gender, a personal psychiatric history and daily time spent on news and events related to COVID-19 on media as predictors of PTSD symptoms. Youngest Tunisian participants reported significantly higher prevalence of PTSD [20]. González et al. noted 15.8% of participants in the Spanish population with PTSD symptoms, with the female gender more susceptible to develop symptoms of PTSD [21]. Zhang and Ma (2020) and N. Liu et al. (2020) reported similar prevalence rates of PTSD symptoms, at 7.6 and 7%, respectively, in the Chinese population [20, 22].

Non-specific psychological distress was also assessed in the Italian population. Ferrucci et al. (2020) investigated, through an on line survey, the intensity and prevalence of self-reported feelings of anxiety, fear, sadness, anger, and concern during the pandemic, and the impact on daily life activities (e.g. nutrition, sleep, sexuality, relationship with others), through ad hoc questions that inquired about the intensity of certain feelings/emotions (scored on a four-point scale 0 = “none” and 4 = “very high” intensity). They found that Italian people felt psychologically vulnerable, females and younger people in particular, because of the imminent economic crises and risk of infection, with a related negative impact on daily life activities, such as sexuality, nutrition, sleep [23]. Young adults are highly exposed to social media and uncontrolled web information, which can trigger psychological distress [24]. Also Mazza et al. (2020) provided a report on the psychological impact and mental health of the general public in Italy during the first peak of the COVID-19 outbreak [25]. Their questionnaire was administered on an online survey platform and disseminated through social networks and emails. The authors included the Depression, Anxiety and Stress Scale–21 items (DASS-21) in their survey, and this research was one of the first attempt to assess the psychological impact of Covid-19 through objective standardized measures. Results showed that female gender, negative affect, and detachment were associated with higher levels of depression, anxiety, and stress. Starting from such evidence, this study aimed to explore the prevalence of post-traumatic stress symptoms and general anxiety symptoms during the first wave of COVID-19 among Italian citizens, by administering standardized tools and by providing a more concrete measure of the mental health situation in the Italian population during the most challenging months of the pandemic. The secondary aim was to correlate socio-demographic risk and protective factors that could affect psychological burdening and the contingent behavioural reactions, such as information seeking. The following contribution does not claim to be included in the psychiatric field and diagnosis of mental disorders, but it is placed in the trend of the exploratory studies investigating the psychological impact of COVID-19 outbreak [20, 26–28]. Such kind of studies may help government and healthcare professionals to prepare targeted interventions and safeguard the psychological well-being of the population against COVID-19 and other outbreaks as well as manage their long-term consequences.

## Methodology

### Procedure

Participants were eligible for the study if they resided in Italy and spoke Italian. They were recruited adopting a snowball sampling method during the COVID-19 quarantine, through a social media message containing the link to the survey. Initially, the link of the survey was mainly shared across different social media accounts and groups (Whatsapp, Facebook, Instagram) belonging to three of the authors (EB, SO, PG). The survey was hosted on Qualtrics, and an anonymous link was generated to be distributed among the population. People landing on the first page of the survey were asked to answer two questions related to their country of residence and their knowledge of the Italian language. If they did not met these inclusion criteria, through automatic filtering of the survey system, they were taken to the last page of the thank you page of the survey.

Snowball sampling is a type of chain-referral nonprobability sampling technique, which involves the recruitment of new participants required for the study by existing participants [29, 30]. The primary data source is selected at the outset of the study and encouraged to invite relatives, friends or acquaintances to take part in the survey. Snowball sampling methods are particularly useful when the study population is unknown, and therefore eligibility criteria cannot be identified. This massive sampling strategy, by the spread of the survey through different social media, is understandable having into account the circumstances of confinement due to COVID-19 outbreak. Finally, demographic information for our participants could not be accessed, and consequently snowball sampling was the ideal sampling method for our study. Recent studies during the COVID-19 pandemic have also successfully used this technique to investigate mental health issues in the general population [20, 26–28, 31].

Participation in the study was voluntary and anonymous and could be withdrawn at any time. The first page of the survey contained the informed consent form, which explained the objective of the study and data handling methods outlined by Italian laws and the European General Data Protection Regulation (GDPR). Participants signed consent forms and completed questions regarding socio-demographic characteristics as well as questions regarding COVID-19 symptoms, information-seeking behaviours, trauma caused by the COVID-19 outbreak, and anxiety symptoms. The questionnaire was available from March 25th to May 1st, 2020, A stay-at-home order was in place during this time, which started three weeks after the partial lockdown (school closures) until the last day of the full lockdown (closure of all non-essential businesses and movement restrictions) in Italy.

### Participants

The survey was accessed by 1455 individuals. Of these individuals, 1195 completed 60% or more of the survey and were considered for advanced analyses. The sample was 71.2% women (*n* = 851), 28.6% men (*n* = 342), and 0.1% not specified (*n* = 2). Mean age was 40 years (s.d. = 14.948). Participants resided in 78 Italian provinces (out of 107 territorial divisions), with 25.7% (*n* = 307) residing in the Milan province and 9.2% (*n* = 110) from the Monza and Brianza provinces. These two provinces were closest to the epicentre of the Italian outbreak.

### Measures

The survey included ad hoc sections and standardized questionnaires. The sections are described below. Socio-demographic information and health status: participants were asked their age (on a continuous scale), gender (male, female, other), education level reached (primary school diploma, middle school diploma, high school diploma, bachelor degree, post-graduate degree, doctoral or similar degree, other), marriage (married/cohabiting, widowed, divorced/separated, single, other) and parental status (‘Do you have children?’ Yes/No), caregiving status of people with immune system diseases (‘Do you care with a person with immune deficits?’ yes/no answer), and presence of chronic diseases (‘Do you have any chronic disease’ Yes/No answer), and immune status (‘Are you immuno-suppressed or immuno-compromised?’ yes/no answer). These last questions have been formulated considering the nature of the COVID-19 viral infection and the scientific reports about mortality rates among people suffering of immune deficiencies [32].

COVID-19 related symptoms: a list of 11 symptoms (plus a blank box to add information) was presented to the participant to assess their physical condition, with a multiple answer option (no limits in the number of selected symptoms). This list of symptoms was included to control if participants’ health status interfered with anxiety and PTSD symptoms. The list was compiled using the symptom list available on the World Health Organization’s (WHO) website [33] at the time of the survey construction (tiredness, anosmia and/or dysgeusia, sore throat, fever, dry cough, shortness of breath, chest pain, diarrhoea, muscle pain, nasal congestions, running nose). It is imperative to remember that, initially, there was only a partial list of symptoms, as compared to the current knowledge regarding the virus. The score for such variable corresponded to the total COVID-19-related number of symptoms selected by the subject.

Participants’ Information-Seeking Behaviour (ISB) was also tested through an ad-hoc questionnaire: participants were presented a list of nine sources of information and asked on a 7-point Likert scale (never to constantly) whether they used it to keep informed about the COVID-19 outbreak. Sources of information included family physicians, a Coronavirus toll-free information line, governmental websites and newspapers, social media, and scientific journals. Final scoring was calculated as an average of all items.

Impact of Event Scale Revised (IES_R) [34] in its validated Italian version [35] was used to assess the trauma symptoms caused by the COVID-19 outbreak and consequent confinement. Some evidence suggested that the IES-R can discriminate between individuals with and without PTSD [20]; however, there is insufficient evidence supporting the IES-R as a specific diagnostic tool. The scale comprises 22 items divided on three subscales corresponding to three dimensions of PTSD diagnosis and rated on a 5-point Likert scale of frequency (1=“not at all”; 5 = “extremely”). The three subscales included the following: Intrusion (8 items, IES_RI), corresponding to the B criterion of PTSD (sample item: “Any reminder brought back feelings about it”); Avoidance (8 items, IES_RA) for the C criterion of PTSD (sample item: “I avoided letting myself get upset when I thought about it or was reminded of it”); and Hyperarousal (6 items, IES_RH) for the D criterion of PTSD (sample item: “I felt irritable and angry”). The total score, which ranges from 0 to 88, provided a measure of the severity of PTSD. There were no specific cut-off scores for the IES-R, though higher scores were representative of greater distress. Increased overall scores on all subscales may indicate the need for further evaluation. There have been several cut-off values suggested for a probable diagnosis of PTSD ranging from 22 to 24 in individuals with substance use disorders [36] and from 33 to 88 in Vietnam veterans [37]. These cut-off values have been based on specific population groups and relate to the raw sum of scores. Reliability for the full scale is good (Cronbach’s alpha = .924); while the IES_RI (alpha = .874), IES_RA (alpha = .767), IES_RH (alpha = .843) have adequate internal consistency.

Generalized Anxiety Disorder scale (GAD-7) [38] in its validated Italian version was used to assess anxiety symptoms. The scale has 7 items for frequency of symptoms over the previous two weeks rated on a 4-point Likert scale (1= “not at all”; 4 = “extremely”). The sample item is “Feeling nervous, anxious, or on edge.” The scale was unidimensional, and the final score was the sum of all the answers. Scores of 5, 10, and 15 were used as the cut-off points for mild, moderate, and severe anxiety symptoms, respectively. Using the threshold score of 10, the GAD-7 had a sensitivity of 89% and specificity of 82% for GAD. The scale has good internal consistency (Cronbach’s alpha = .908).

### Analytic strategy

Analyses were performed on IBM SPSS v25. Raw scores for GAD-7 and dimensions of IES-R were first calculated. COVID-19 related symptoms were summed up and the raw count coded into quartile groups. Age was grouped according to generation (Baby Boomer: born between 1946 to 1965; Generation X: born between 1966 to 1980; Generation Y: born between 1981 to 1995; and Generation Z, born between 1996 to 2001). Parametric MANOVA and ANOVA were applied to compare groups of subjects for quantitative continuous variables, such as IES-R and GAD-7 scores. Non-parametric analyses Kruskal-Wallis’ test (H) and Mann-Whitney’s Test (U) were preferred when variable scores distribution did not satisfy normality and homogeneity variance distribution criteria, or groups identified by socio-demographic factors did not have a comparable sample size. Spearman’s coefficient was calculated to correlate the number of COVID-19 related symptoms reported by the participants or the levels of Information Seeking Behaviour with the IES-R and GAD-7 scores.

## Results

The total IES_R mean score for the whole sample was 27.86 (s.d. = 17.46) (index of high PTSD levels if considering the cut-off in other populations) [36] with a highest score of 81 (out of 88), while the mean score for the GAD-7 was 6.89 (s.d. = 5.08) (index of mild general anxiety disorder), with a highest score of 21 (out of 28).

Table 1 shows non-parametric test results for the following independent variables: gender, presence of chronic disease, immunosuppressed/immunocompromised status, education level, marriage status, and parental status.

**Table 1:** Non-parametric test results for socio-demographic variables and the IES-R dimensions and the GAD-7

	N(%)	IES_RI Mdn (IQR)	IES_RA Mdn (IQR)	IES_RH Mdn (IQR)	GAD-7 Mdn (IQR)
Gender^a^					
Male	342(28.6)	6.00(8.00)^**^	6.00(7.00)^**^	4.00(7.00)^**^	4.00(6.00)^**^
Female	851(71.2)	10.00(9.00)^**^	10.00(9.00)^**^	8.00(8.00)^**^	7.00(7.00)^**^
Chronic Diseases^b^					
Yes	234(19.58)	10.00(10.00)	10.00(8.00)	8.00(9.00)	6.50(8.00)
No	961(80.41)	9.00(9.00)	9.00(9.00)	7.00(9.00)	6.00(6.00)
Immunosuppressed/Immunocompromised					
Yes	59(5.38)	11.00(8.00)^*^	10.00(7.75)	8.00(7.00)	7.00(8.00)
No	1036(94.61)	9.00(9.00)^*^	9.00(9.00)	7.00(9.00)	6.00(7.00)
Caring for a person with immune deficits^a^					
Yes	139(11.63)	10.00(10.25)	9.00(10.00)	8.00(9.00)	7.00(7.00)
No	1056(88.61)	9.00(9.00)	9.00(9.00)	7.00(8.75)	6.00(7.00)
Education^a^					
Primary	4(0.36)	NA	NA	NA	NA
Middle School	68(6.21)	9.00(10.00)	9.00(10.00)	6.00(7.50)	4.00(7.75)
High School	457(41.73)	10.00(10.00)	10.00(8.00)^**^	7.00(9.00)	6.00(7.00)
Bachelor	109(9.95)	9.00(9.00)	9.00(8.00)	8.00(9.00)	7.00(8.00)
Postgraduate (MS, …)	291(26.57)	10.00(10.00)	9.50(8.00)^**^	7.00(8.00)	6.00(6.00)
Doctoral or similar	166(15.15)	8.00(7.75)	8.00(6.00)^**^	6.00(7.75)	6.00(6.00)
Marital Status^b^					
With partner		9.00(10.00)	9.00(8.00)*	7.00(8.00)	5.00(6.00)*
With no partner		9.00(9.00)	10.00(8.00)*	7.00(8.00)	6.00(7.00)*
Other	89(7.49)	8.00(9.00)	9.00(8.00)	6.00(7.00)	8.00(5.50)^*^
Parenthood^b^					
Yes	458(38.32)	9.50(9.00)	8.50(8.00)^*^	6.50(7.00)^*^	5.00(6.50)
No	737(61.67)	9.00(9.00)	10.00(8.00)^*^	7.00(8.00)^*^	6.00(7.00)

Notes: * indicates *p* < .05; ** indicates p < .001; ^a^ refers to Kruskal-Wallis’ test H; ^b^ to Mann-Whitney’s Test U

Gender differences for the three dimensions of the IES-R (IES_RI: H (1) = 71.232, *p* = .001; IES_RA: H (1) = 61.289, p = .001; IES_RH: H (1) = 67.348, p = .001) and GAD-7 (H (1) = 82.919, p = .001) resulted to be significant, with women obtaining consistently higher median scores than men in all IES-R subscales and GAD-7.

Individuals with a chronic disease did not yield different results than those without a chronic disease in all considered variables. Individuals with immunocompromised or immunosuppressed status significantly differed in median scores (mdn = 11.00, IQR = 8.00) from individuals without any immune-deficit condition (mdn = 9.00, IQR = 9.00) for Intrusion scale only (IES_RI) (U = 20,527, z = − 2.679, *p* = .007); however, they did not significantly differ on the other dimensions of IES scale and GAD-7. Participants who were caregivers for immunocompromised individuals did not significantly differ for any of the considered variables, although the Intrusion subscale showed a slight trend toward significance (IES_RI; U = 53,209, z = − 1.83, *p* = .066). Education level significantly affected the Avoidance symptoms (IES_RA; H (5) =16.514, *p* = .006). Pairwise comparisons, with Bonferroni error correction, revealed that the significant difference was between participants with doctoral or similar level of education (mdn = 8.00, IQR = 6.00) and participants with an high school diploma (mdn = 10.00, IQR = 8.00), or with a bachelor degree (mdn = 9.50; IQR = 8.00). Thus, people with a higher educational levels showed to be less avoidant than the other groups. The presence of a partner/sentimental relationship also provided significant results for the Avoidance dimension of IES_R (U = 122,473.00, z = 2.101, *p* = .36) and GAD-7 (U = 121,491.50, z = 2.042, *p* = .041). More specifically, individuals with partners had significantly lower median scores than individuals who were single both in IES_A (mdn = 9.00, IQR = 8.00 vs mnd = 10.00, IQR = 8.00) and GAD (mdn = 5.00, IQR = 6.00 vs mdn = 6.00, IQR = 7.00).. Participants with children (Parenthood) had significantly lower levels of Avoidance (IES_RA; U = 145,029.50, z = 2.536, *p* = .011) and Hyperarousal (IES_RH; U = 142,434.00, z = 2.001, *p* = .045) compared to non-parents (IES_RA mdn = 8.50, IQR = 8.00 vs mdn = 10.00, IQR = 8.00; IES_RH mdn = 6.50, IQR = 7.00 vs mdn = 7.00, IQR = 8.00).

A MANOVA was used to test differences among generations. Using Pillai’s trace, a significant effect of belonging to a specific generation on the Impact of Event Scale was found (V = .746, F (3,1041) =1017.19, *p* = .001). Post-hoc analysis with separate univariate tests on the IES dimensions revealed non-significant differences for Intrusion (IES_RI) and Avoidance (IES_RA) among the different generations. Instead, the Hyperarousal dimension of the IES-R was significant (IES_RH; f (3,1043) = 3.112, *p* = .026) with Generation Y (m = 8.37, s.d. = 5.68) showing higher levels of hyperarousal compared to Baby Boomers (m = 6.98, s.d. = 4.80).

An univariate ANOVA was used to test differences among generations on general anxiety symptoms (GAD-7). Significant results emerged in GAD-7 scores among three generations (F (3,1041) = 5.113, *p* = .002). Particularly, post-hoc analyses revealed that Baby Boomers (m = 5.69, s.d. = 4.74) had significantly less anxiety symptoms than Generation Y (m = 7.38, s.d. = 5.19) and Generation Z (m = 7.42, s.d. = 5.07), indicating overall higher levels of anxiety symptoms in the younger generations.

Table 2 shows correlations between the IES dimensions, GAD-7, and Covid-19-related number of symptoms, and the Information-Seeking Behaviours for different information sources. It is important to note that the Covid-19-related number of symptoms positively correlated with all variables considered, whereas the Information-Seeking Behaviours differently correlated with the other scales based on the information sources. It is worth noting that information seeking on social media positively correlates with all IES-R subscales and GAD-7; information seeking through direct contacts instead (e.g., phone calls with family physicians or to the Coronavirus toll-free information line) is only correlated to the Avoidance dimension of IES-R. Information seeking through traditional media (e.g. newspapers) positively correlated with the Intrusion dimension (IES_RI), and information seeking through institutional sources (e.g. national and regional websites) positively correlated with GAD-7 and all IES_R dimensions, except for Avoidance.

**Table 2:** Correlation analyses for number of Covid-19-related symptoms, levels of Information Seeking Behaviour, and the IES-R dimensions and GAD-7

Covid-19-relatedsymptoms	IES_RI	IES_RA	IES_RH	GAD-7
	.145**	.112**	.217**	.247**
Information-Seeking Behaviours				
Social Media	.222**	.138**	.202**	.213**
Traditional Media	.140**	.005	.054	.048
Institutional Sources	.133**	.029	.128**	.094**
Direct Contact	.014	.075*	−.002	−.017

Notes: ** Spearman’s correlation is significant at .01. (two-tailed). * Spearman’s correlation is significant at .05 (two-tailed)

## Discussion

Panic and anxiety are recurring elements in the early stages of all crisis contexts, including during a pandemic. In the Italian population, feelings of loss of control and of being forcefully confined have been inevitably exacerbated by the narrative of increased number of cases detected and associated deaths along with a daily bulletin frequently compared to a war bulletin. The greatest consequence of the mandatory lockdown was fear (of being infected, of infecting children or elderly parents, and of economic crisis) [39–44] which is strongly associated with mental health [43]. Results from this exploratory study seem to suggest partial agreement with finding from emerging research about COVID-19 and especially the reviews trying to collect evidences from different countries, e.g. Hossain et al. [17, 18], which identifies gender, age, marital status, education, and health problems as factor impacting mental health during the current outbreak and other infective diseases. Our data showed that the Italian population felt shocked by worsening of the pandemic and the resulting lockdown to reduce its spread, with average scores of PTSD symptoms nearing the threshold for a full diagnosis of the disorder, when referring to cut-off values set for other populations (cut-off ranging from 22 to 24 in individuals with substance use disorders) [36]. The overall level of symptoms of anxiety exceeded the cut-off expected for “mild anxiety” (cut-off for mild anxiety in the GAD scale is 5, and the mean score for the population was 6.89), showing a high risk of developing anxiety disease.

Gender appeared to play an important role in developing PTSD and anxiety symptoms in the Italian sample involved in this study. Women displayed higher level of anxiety (m = 7), which is close to the cut-off point of 8 found in clinical populations [38]. In a population-wide catastrophe, women react with higher levels of distress: they tend to ruminate on the event (Intrusion) and attempt to avoid the issue (Avoidance) yet still develop symptoms linked to generalized anxiety (Hyperarousal). These results confirm previous literature that women are more psychologically and emotionally at risk during a traumatic event [45], including during the COVID-19 outbreak [46].

In the Italian self-selected sample considered in this study, participants with chronic diseases do not seem to show a higher likelihood of developing PTSD or GAD symptoms, as reported in other traumatic events [47]. This result is not coherent with other studies that focused on emotional reactions of populations with different chronic diseases during the COVID-19 pandemic [48–50]. Ozamiz-Extebarria et al. (2020) reported that, in Spain, younger individuals with chronic diseases complained more of symptoms related to anxiety and depression during the arrival of the virus and consequent confinement than the rest of the population [49]. Additionally, the presence of a chronic disease as a risk factor for anxiety and depression during the COVID-19 pandemic has been observed by Ping et al. (2020, 29] in the general population in China and by Salari et al. (2020) in patients with Parkinson’s Disease [48] and Epilepsy [51] in Iran. Participants with immunocompromised or immunosuppressed status were an exception in our sample as these individuals have more frequent intrusive symptoms than those who are not immune-deficient. Nature, duration, and a cure for COVID-19 are unknown; therefore, those with this status may feel more at risk of contracting the virus and may be consumed by a circular thought process of self-protection, fear of contagion, and concern for caregivers [52]. The lack of coherent significant results on immunocompromised or immunosuppressed patients may be due to the severity of the chronic illness, which was not observed and analysed in this study. Caregivers do not have a significantly different risk of PTSD or GAD symptoms from the rest of the population in the Italian sample examined, although there is emerging evidence that they may experience more distress than both patients and the general population [17, 53]. Higher educational levels appear to be a protective factor in the sample analysed, specifically in regards to tendency to avoid information about the traumatic event. These results seem to only partially confirm the results obtained in other studies both on the Italian population [23, 25] and populations from other countries [14, 54], which observe clearer effects of educational levels on mental health. It is possible to hypothesize that a higher level of education implies more ability to understand information about the pandemic and to manage the complexity behind the situation, thus curbing anxiety and traumatic responses. Having a partner seems to be a protective factor against anxiety, and the Italian sample appears to support this finding from other studies [54]. Having someone to lean on during the pandemic may also ease the burden of uncertainty caused by the situation. Another socio-demographic characteristic that appears to have a mitigating effect on psychological distress is parenthood. Parents develop less symptoms of PTSD (Avoidance and Hyperarousal) than the rest of the population. This is likely due to the fact that efficient caregiving for their children would not allow a complete focus on themselves and would discourage avoidance and excessive reactions. Generational differences (thus age differences) highlighted in this research are aligned fully with other COVID-19-related findings [14, 22, 25, 54, 55] that suggest that younger generations are more vulnerable to different dimensions of psychological distress than older generations. Younger individuals are least prepared to face trauma or may be more informed about the nature of Coronavirus than their older counterparts, which may generate more anxiety and PTSD symptoms due to fear for themselves, their parents, and/or their offspring. Furthermore, the COVID-19 crisis presents sizable risks among younger generations in the areas of education, employment, mental health, and disposable income. These younger generations will heavily carry the long-term economic and social burdens secondary to the pandemic, which may ultimately impact their overall health and well-being as they focus primarily on short-term economic and equity resolutions.

Finally, a more intense information-seeking behaviour (especially for mediated sources of information) correlates highly with symptoms of PTSD and anxiety, confirming previous findings in other populations [43, 46]. Feelings of uncertainty and general lack of clear information about the virus may trigger seeking more information, whose quality and quantity may cause increasing levels of distress [43, 56].

### Limits

While the results are based on a large sample, the results are limited by the sampling technique, which does not allow to generalize the considerations made here. As seen in Table 3, our sample is slightly divergent from the general population, with female, and highly educated people being over represented, while people with chronic disease being under-represented, but our contribution is in line with other recent studies [23, 25] for both sample composition and directions of results.

**Table 3:** Frequencies and percentages for the socio-demographic characteristics of the sample and in the Italian population

	Sample		Italian Population (over 18)	
	N	%	N	%
Gender^a^				
Male	342,00	28,62	29,340,565,00	48,18
Female	851,00	71,21	30,904,074,00	51,82
Other	2,00	0,09		
Total	1195,00	100,00	60,244,639,00	100,00
Generation^b^				
Baby Boomers	315,00	26,40	15,350,381,00	35,21
Gen X	325,00	27,20	10,120,605,00	23,21
Gen Y	263,00	22,00	13,962,868,00	32,02
Gen Z	292,00	24,40	4,166,245,00	9,56
Total	1195,00	100,00	43,600,099,00	100,00
Education level^a^				
No title			4,920,233,00	8,77
primary	4,00	0,30	11,279,166,00	20,10
Middle School	68,00	5,70	16,706,879,00	29,77
High School	457,00	38,20	16,950,936,00	30,20
BA/BS	109,00	15,50	1,579,856,00	2,81
Post-graduate	291,00	24,40	4,691,104,00	8,36
Doctoral or similar	166,00	13,90		
Total	1195,00	98,00	56,128,174,00	100,00
Chronic Disease^c^				
Yes	234,00	19,58	24,000,000,00	42,76
No	961,00	80,42	32,128,174,00	57,24
Total	1195,00	100,00	56,128,174,00	100,00

^a^ Data from the Italian National Institute of Statistics (ISTAT), calculated on the total of the Italian population based on 2011 census; b Data from ISTAT, calculated on Italian population aged 18–74 based on 2011 census; ^c^ Data from the Italian Istituto Superiore di Sanità’s chronic disease 2019 report

With a snowball sampling technique, self-selection bias due to non-coverage of different population groups (e.g. vulnerable population, people with no access to the Internet) is possible [57]. Since representativeness is not guaranteed, there is not certain knowledge os the distribution of the population. Sampling bias also may affect the results, because the link to the survey may be passed from participants to people they know well, thus possibly obtaining a sample of participants that share similar characteristics. This obviously limits the scope of the hypothesis here presented and may bring to biased estimates [58].

Finally, post-traumatic stress symptoms and depression commonly co-occur [59]. Some symptoms of depression and PTSD overlap, but they remain two distinct trauma-related phenotypes [60]. Nevertheless, in order to avoid burdening the participants, only a few measurements of interest were selected, specifically focused on PTSD symptoms and anxiety, typical of the acute reaction to trauma [61, 62], avoiding to add a further questionnaires to measure other symptoms, such as depression, rumination, etc.

## Conclusions

The COVID-19 pandemic of 2020 has not only had a physical impact, as Italian citizens appear to be experiencing ever-increasing levels of distress due to the uncertainty and the limitations posed by the pandemic. While negative psychological reactions are to be expected [63], it is important to act swiftly to avoid a psychological crisis in which symptoms of PTSD and GAD actually develop into full-blown disorders. It is also fundamental that future research is carried out to track the mental well-being of the general population throughout the entire duration of the pandemic- Another major focus should also be vulnerable groups (i.e., individuals with cancer or immunosuppressed, and younger generations) who may be more strongly impacted by the pandemic. For instance, to avoid exacerbating intergenerational inequalities and to involve young people in building societal resilience, we need to anticipate the psychological impact of the pandemic across different age groups through application of effective monitoring. Eliminating Fear of COVID-19 [42] – and of future diseases – is paramount. Preventative interventions targeting mental health should be in place for any country-wide emergencies and should also be part of the intervention packages deployed by governments. Anecdotal evidence shows that Italy is slowly addressing the psychological side of the pandemic. For example, in the midst of the pandemic, the regional government of Campania (one of the most populous areas of Italy) ruled to have a “family psychologist,” a figure similar to the family physician but focused on individuals’ mental health, as mental health has been becoming an increasingly more important concern for people and institutions. The results presented here aim to help identify particularly vulnerable populations, like women and younger generations, who should be among the first recipients of mental health care from Public Health officials. Addressing mental health during a crisis such as the COVID-19 one is important because individuals affected by mental health issues may have less resources to understand and apply preventative behaviours that may help prevent the spread of the pandemic [2] and also important consequences, such as suicide, which is already a phenomenon registered during this pandemic [64–66]. Monitoring and intervening on mental health of the population and especially of vulnerable groups would also help avoiding the chronicization of the symptoms into full-blown psychiatric diseases that may affect people not just during, but also after the outbreak, imposing new, raising costs on health systems that have already been strained by the pandemic.

## Data Availability

The raw data supporting the conclusions of this article will be made available by the authors upon request, without undue reservation.

## Abbreviations

COVID-19: 2019/2020 SARS-CoVID Coronavirus disease
PTSD: Post-traumatic Stress Disorder
GAD: Generalized Anxiety Disorder
IES-R: Impact of Event Scale – Revised
IES-RI: Impact of Event Scale – Revised, Intrusion dimension
IES-RH: Impact of Event Scale – Revised, Hyperarousal dimension
IES-RA: Impact of Event Scale – Revised, Avoidance dimension
GAD-7: Generalized Anxiety Disorder Scale – 7 items
